# Anogenital distance is related to ovarian follicular number in young Spanish women: a cross-sectional study

**DOI:** 10.1186/1476-069X-11-90

**Published:** 2012-12-08

**Authors:** Jaime Mendiola, Manuela Roca, Lidia Mínguez-Alarcón, Maria-Pilar Mira-Escolano, José J López-Espín, Emily S Barrett, Shanna H Swan, Alberto M Torres-Cantero

**Affiliations:** 1 Division of Preventive Medicine and Public Health, Department of Health and Social Sciences, University of Murcia School of Medicine, Espinardo Campus, Espinardo (Murcia), 30100, Spain; 2 Fertilidad Roca, Gestión Clínica Avanzada SLU, Avenida Ronda Sur 20, Murcia, 30010, Spain; 3 Center of Operations Research, Miguel Hernandez University, Elche Campus, Elche, 03202, Spain; 4 Department of Obstetrics and Gynecology, School of Medicine and Dentistry, University of Rochester, 601 Elmwood Avenue, Rochester (NY), 14624, USA; 5 Department of Preventive Medicine, Mount Sinai School of Medicine, 1 Gustave L. Levy Place, New York (NY), 10029, USA; 6 Regional Campus of International Excellence "Campus Mare Nostrum", University of Murcia, Avenida Teniente Flomesta 5, Murcia, 30003, Spain

**Keywords:** Androgens, Anogenital distance, Increased follicular recruitment, Ovarian morphology

## Abstract

**Background:**

In animals, anogenital distance (AGD) at birth reflects androgen levels during pregnancy and predicts adult AGD. Little is known about AGD in relation to female reproductive characteristics in humans, a question this study was designed to explore.

**Methods:**

We used multiple linear and logistic regression analyses to model the relationships between adult female reproductive system characteristics (e.g. ovarian morphology, menstrual cycle) and two measures of AGD [anus-fourchette (AGD_AF_) and anus-clitoris (AGD_AC_)] in 100 college-age volunteers in Spain. Ovarian morphology was classified as having < 6 or ≥ 6 follicles per ovary.

**Results:**

Both AGD measures were positively associated with ovarian follicle number, with AGD_AF_ being more strongly associated. Women in the upper tertile of the AGD_AF_ and AGD_AC_ distributions were more likely to have ≥ 6 ovarian follicles [OR: 6.0 (95% CI 2.0, 17.6) and 3.0 (95% CI 1.1, 8.6), respectively] compared to women in the lowest tertile.

**Conclusions:**

Increased follicular recruitment has been related to excess androgen exposure in utero in toxicological studies. Our results suggest that the androgenic environment during early fetal life may influence reproductive system development, including AGD, in human females.

## Background

Anogenital distance (AGD) is routinely used as a developmental endpoint in animal toxicology studies by the U.S. Environmental Protection Agency and is one of the most sensitive markers of in utero exposures to environmental endocrine-disrupting chemicals (EDC) [1,2]. A number of animal studies have shown that exogenous androgen or estrogen exposure (including EDC) during the prenatal period can alter the development of the female reproductive tract [3-15]. For example, bisphenol A (BPA), which may have both estrogenic and anti-androgenic properties, has been shown to disrupt and alter ovarian function [10-14]; and it has recently been demonstrated that BPA alters early oogenesis and follicle formation in the fetal ovary of nonhuman primates [14]. Moyer and Hixon [15] have recently shown that prenatal exposure to another well-known EDC, di(2-ethylhexyl) phthalate, increased the numbers of mature follicles in adult females in the highest exposure group and the subsequent decrease in overall reproductive lifespan. In rodents, AGD reflects the amount of androgen to which a female fetus is exposed in early development. Indeed, prenatal exposure of females to exogenous androgens results in longer and more masculine AGD [5-7] and increased ovarian follicular recruitment in adulthood compared to controls [3,4,7]. In male rodents, shortened (weight-adjusted) AGD persists into adulthood [16] and predicts compromised reproductive function in the mature male [17,18]. Therefore, in rodents AGD appears to provide a life-long read-out of prenatal androgen action [17].

Swan and colleagues showed a strong inverse association between prenatal environmental exposure to the anti-androgenic phthalates and shorter male AGD in human infants [19,20]. Recently, several studies on adult men have provided strong evidence of the relationship between AGD length and male reproductive function [21,22]. Thus, AGD may provide a reliable link between prenatal hormonal milieu and adult reproductive function in human males as well. Less is known about AGD in human females, but there are several published reports of virilization of the lower urogenital tract after either prenatal exogenous or endogenous exposure to androgenic hormones [23,24]. Although a number of studies have measured female AGD measurements in human infants [19,20,23,25-27], to our knowledge, no study has measured AGD in adult women. The aim of this study is to investigate the relationship between AGD measures and adult female reproductive system characteristics.

## Methods

### Study population

The Murcia Young Women’s Study (MYWS) is a cross-sectional study of healthy young university students (18–23 years old) in the Murcia region of Spain. MYWS was carried out between 9^th^ February 2011 and 25^th^ November 2011. Written informed consent was obtained from all subjects. The Research Ethics Committee of the University of Murcia approved this study.

Flyers stating, “Young healthy female university students wanted for research project” were posted at university campuses to invite students to participate in this study. To be included in MYWS, subjects had to be university students, have been born in Spain after 31^st^ December 1987, and be able to contact their mother and ask her to complete a questionnaire. One hundred and twenty-four students contacted us, 15 subjects did not meet inclusion criteria (3 had not been born in Spain and 12 were born before 31^st^ December, 1987), leaving 109 (88%) eligible students of whom 100 (92%) agreed to participate in the study. At a scheduled clinic visit subjects underwent a gynecological examination, including transvaginal ultrasound, and completed an epidemiological questionnaire on lifestyle. Participants were compensated for their participation (€40 gift card).

### Physical examination and gynecological history

Body weight and height were measured using a digital scale (Tanita SC 330-S, London, UK). Body mass index (BMI) was calculated as weight in kilograms divided by squared height in meters. Ovarian and uterine morphology were studied by transvaginal ultrasound using a single ultrasound machine for all imaging studies (Voluson E8®; General Electric Healthcare, USA). Women were scanned in the early follicular phase (cycle days 1–6) by two gynecologists (75% by M.R. and 25% by C.M.) using the same classification.

Uterine morphology was assessed as normal or abnormal. Ovaries were classified as having < 6 or ≥ 6 follicles per ovary [28-30], a cutoff which several studies suggest may have clinical relevance [28,29]. All follicles fell in the 2–10 mm diameter range. Having 6 or more follicles has been associated with hyperprolactinaemia, hypothalamic anovulation or weight-related amenorrhoea and may result from incomplete pulsatile gonadotrophin (GnRH) stimulation of ovarian follicular development [28]. It may also indicate an ovarian dysfunction in female adolescents with cystic fibrosis [29] or the presence of polycystic ovary [30].

In addition, a complete gynecological history was obtained from each subject, including history of gynecological diseases (salpingitis, endometriosis, other) (yes/no), self-reported menstrual cycle length (days) and previous irregular menstrual cycle (yes/no).

### Anogenital measurements

**Figure 1:**
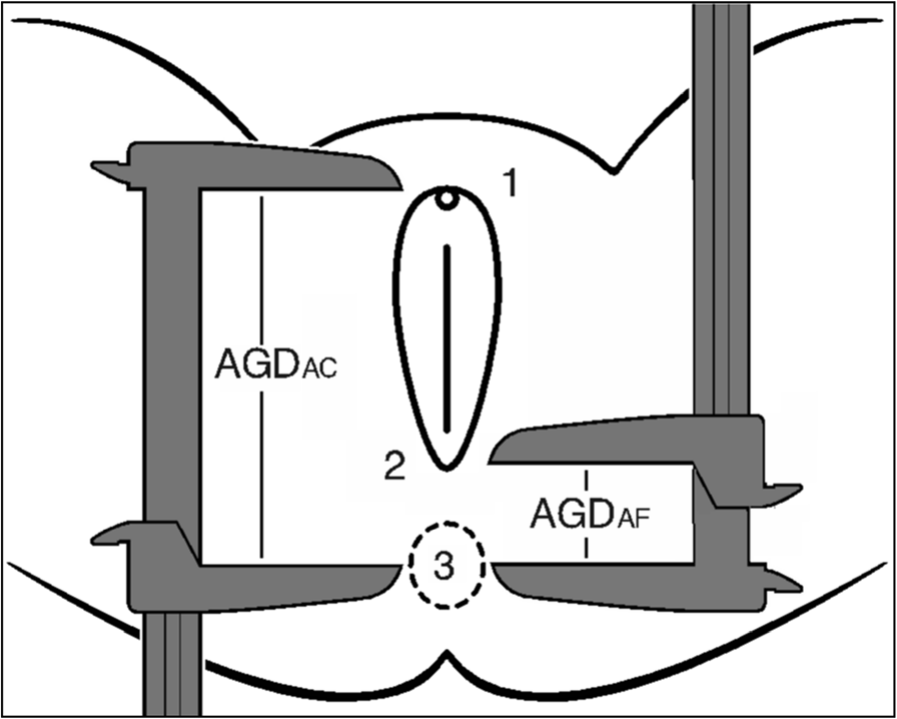
**Landmarks for two measurements of AGD: AGD**_**AC**_**, from the anterior clitoral surface to the center of the anus (point 1 to point 3); and AGD**_**AF**_**, from the posterior fourchette to the center of the anus (point 2 to point 3).** Adapted with permission from Sathyanarayana et al. [27].

For each subject, AGD was measured in two ways. First, AGD_AC_ was measured from the anterior clitoral surface to the center of the anus (Figure 1, point 1 to point 3). Second, AGD_AF_ was measured from the posterior fourchette to the center of the anus (Figure 1, point 2 to point 3). Measurements were made using a digital caliper (Stainless Steel Digital Caliper, VWR® International, LLC, West Chester, PA, USA) and while subjects were in the lithotomy position, with the thighs at a 45° angle to the examination table. To improve precision, two examiners made each of these measurements three times, taking in total 6 measures for AGD_AF_ and AGD_AC_, respectively. The mean of the six measurements was used as the estimate. Neither the examiners nor the support staff had knowledge of any of the women’s possible conditions/alterations because the AGD measures were taken before the gynecological examination.

### Statistical analyses

We assessed intra- and inter-examiner variability in the AGD measurements by calculating the coefficient of variation (% CV). We used multiple linear regression analyses to identify predictors of each of the two AGD measurements. Covariates initially examined as predictors of AGD measurements were: age, height, weight, body mass index (BMI; kg/m^2^), age at menarche, self-reported sexually transmitted diseases (STD), taking any medication (antibiotics or antihistamines; yes/no) and hormonal contraception (yes/no). When inclusion of a potential covariate resulted in a change in the β - coefficient of < 10%, the variable was not retained in final models. Adjusted odds ratios (ORs) and 95% confidence intervals (CIs) were calculated using logistic regression. All tests were two-tailed and the level of statistical significance was set at 0.05. Statistical analyses were performed with the statistical package IBM SPSS 20.0 (IBM Corporation, Armonk, New York, USA).

## Results

Table 1 shows the general characteristics of young women attending the MYWS. The MYWS study population was quite homogeneous. Participants were nulliparous, mostly in good or excellent health, with neither self-reported STD, nor injuries in the genital region, nor transfusions. None of the women had been diagnosed with salpingitis or endometriosis. Only one woman presented abnormality of the uterus (bicornuate uterus). Sixty-five percent of the young women reported having ever had irregular menstrual cycles. In terms of ovarian morphology, 43% of the women presented < 6 ovarian follicles and 57% ≥ 6 ovarian follicles. Women with < 6 follicles had a mean [± standard deviation (SD)] AGD_AF_ of 35.3 (5.3) mm and AGD_AC_ of 77.3 (9.7) mm. Women with ≥ 6 follicles had a mean AGD_AF_ of 39.4 (6.5) mm and AGD_AC_ of 82.6 (10.7) mm.

**Table 1:** **Characteristics of young women participating in the Murcia Young Women’s Study (MYWS)**^**(1)**^

	**Mean (SD)**	**Median (5–95)**
Age (years)	20.0 (1.2)	20.0 (18.0-22.0)
BMI (kg/m^2^)	21.8 (3.1)	21.3 (17.7-28.8)
Age at menarche	12.7 (1.3)	13.0 (11.0-15.0)
Day of cycle at clinic	3.9 (2.6)	3.5 (1.0-6.0)
Menstrual cycle length^a^	30.9 (7.6)	30 (25.0-44.0)
Anogenital distance (AGD_AC_) (mm)	80.4 (10.5)	79.2 (59.5-96.1)
Anogenital distance (AGD_AF_) (mm)	37.7 (6.3)	37.2 (27.9-48.6)
AGD_AF_ / AGD_AC_	0.47 (0.09)	0.46 (0.36-0.60)
	Percentage of women (%)	
Caucasian	98.0	
Cigarette smoking^b^	34.0	
Alcohol intake (liquor)^c^	31.6	
Have had:		
Good or excellent general health^d^	92.0	
Diabetes or thyroid disease	2.0	
Using hormonal contraception^e^	39.8	

^1)^ One woman with no physical examination performed (n = 99).

^a)^ in days (number of subjects = 36).

^b)^ Mean number ± (SD) of cigarettes per week, 39.4 ± (29.9), if a smoker.

^c)^ Mean number ± (SD) of drinks (330 cc) per week, 2.6 ± (2.5), if a drinker.

^d)^ Question was ‘How would you describe your own health?

^e)^ Oral contraceptives or vaginal ring.

SD = Standard deviation; (5–95) = 5^th^-95^th^ percentile.

AGD_AC_: Anogenital Distance from the center of the anus to the anterior clitoral surface.

AGD_AF_: Anogenital Distance from the center of the anus to the posterior fourchette.

**Figure 2:**
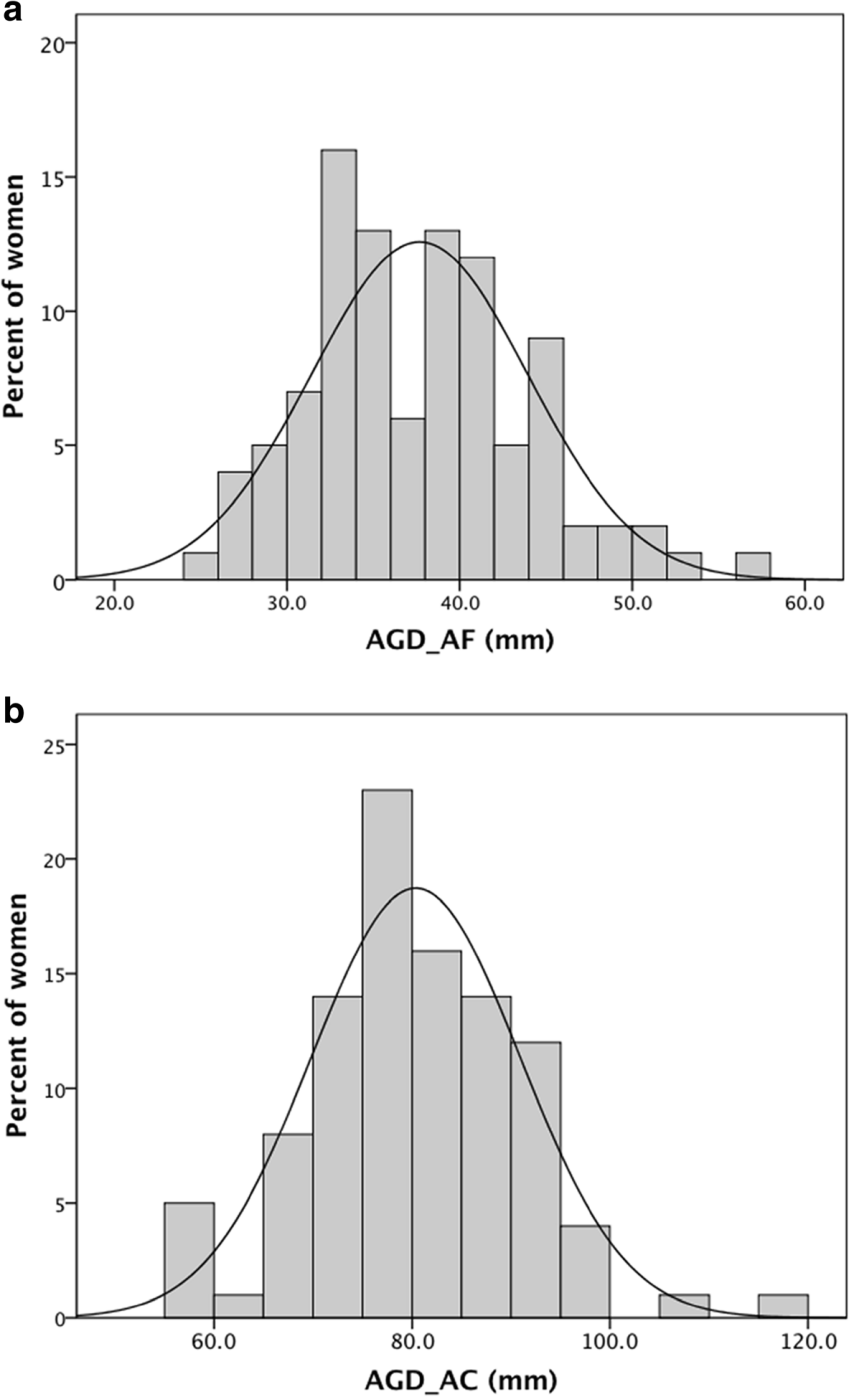
**Frequency distributions of (a) AGD**_**AF **_**and (b) AGD**_**AC **_**in the MYWS**.

**Figure 3:**
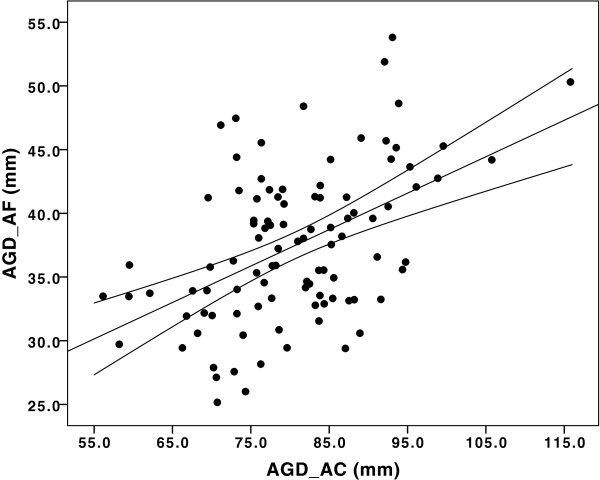
**Correlations between AGD**_**AF **_**and AGD**_**AC **_**measures in the MYWS**.

The distributions of both AGD_AC_ and AGD_AF_ were approximately normal (Figure 2a, b). As expected, AGD_AC_ and AGD_AF_ were correlated [Pearson correlation (r) = 0.44, p < 0.0001] (Figure 3). In the multivariate analyses, there was a significant positive association between BMI and both AGD measures (both p < 0.01). Hormonal contraception was associated with shorter AGD measurements (both p < 0.05) (Table 2). None of the other covariates were significantly associated with either AGD measurement.

**Table 2:** **Variables related to AGD**_**AF **_**and AGD**_**AC **_**in multivariate models**

**Variable**	**AGD**_**AF**_			**AGD**_**AC**_		
	**Unstandardized Coefficients**	**Standardized Coefficients**	**P-value**	**Unstandardized Coefficients**	**Standardized Coefficients**	**P-value**
	β	β		β	β	
BMI (kg/m^2^)	0.82	0.41	< 0.01	1.2	0.34	< 0.01
Hormonal contraception^a^	- 2.4	- 0.20	0.03	- 4.9	- 0.24	0.01

^a^ no vs. yes.

As seen in Table 3, both AGD_AF_ and AGD_AC_ were positively associated with the presence of ≥ 6 ovarian follicles. We also examined the association between having < 6 or ≥ 6 ovarian follicles and both AGD measurements stratified in tertiles (33 women in each group). Both AGD measures were significantly associated with having ≥ 6 ovarian follicles after controlling for the same covariates used in the linear regression models. Women with AGD in the upper tertile of the AGD_AC_ distribution, compared to the lowest tertile were more likely to have ≥ 6 ovarian follicles compared to < 6 follicles (Odds ratio: 3.0, 95% CI 1.1, 8.6). Similarly, women in the middle and upper tertiles of the AGD_AF_ distribution, compared to the lowest tertile, were three and six times more likely to have ≥ 6 ovarian follicles compared to < 6 follicles [Odds ratio: 3.2 (95% CI 1.1, 8.8) and 6.0 (95% CI 2.0, 17.6), respectively]. Neither AGD measurement was statistically significantly associated with any of the other reproductive outcomes.

**Table 3:** **Multivariate analysis for young women’s female reproductive system characteristics and AGD**_**AF **_**and AGD**_**AC**_^**(1)**^

**Variable**	**AGD**_**AF**_				**AGD**_**AC**_			
	**β**	**95% CI**	**P-value**	**R**^**2**^	**β**	**95% CI**	**P-value**	**R**^**2**^
Uterine morphology^a^	- 2.0	(− 9.7, 5.7)	0.60	0.22	1.0	(− 12.6, 14.6)	0.88	0.18
Ovarian morphology								
< 6 ovarian follicles	Ref.				Ref.			
≥ 6 ovarian follicles	3.1	(0.96, 5.3)	< 0.01	0.30	4.6	(0.76, 8.5)	0.02	0.23
Menstrual cycle length^b^	0.25	(− 0.24, 0.74)	0.31	0.20	0.46	(− 0.62, 1.5)	0.39	0.23
Irregular menstrual cycle^c^	0.69	(− 1.6, 2.9)	0.55	0.22	0.71	(− 3.3, 4.8)	0.73	0.18

^1)^ Controlling for BMI and hormonal contraception.

^a)^ normal/abnormal.

^b)^ in days (number of subjects = 36).

^c)^ Question was: “Have you ever had an irregular menstrual cycle?” (yes/no).

β = Regression coefficient; CI = Confidence interval.

Ref.: reference group.

### Within and between-examiner variability

Intra-examiner coefficients of variation for both AGD measurements were below 3%, and inter-examiner coefficients of variation for AGD_AC_ and AGD_AF_ were 4.9% and 10%, respectively. Using a mixed model, the interclass correlations were 0.84 (95% CI 0.76, 0.89) and 0.61 (95% CI 0.41, 0.74) for AGD_AC_ and AGD_AF_, respectively.

## Discussion

This is the first study to measure AGD in adult women and examine the relationships between AGD and female reproductive system characteristics. Both AGD measures were positively and strongly associated with the presence of greater ovarian follicular number. Moreover, a woman with AGD_AC_ or AGD_AF_ in the highest tertile of the distribution was 3 and 6 times, respectively, as likely to have ≥ 6 ovarian follicles as a woman with an AGD_AC_ or AGD_AF_ in the lowest tertile. This underscores the possible clinical implications of the associations that we are reporting here.

Although we typically find that in men AGD measurements are not sensitive to physiologic and lifestyle factors [22], in the current study population the use of hormonal contraception was associated with shorter measures in both AGD measurements (p values <0.05). One immediate consequence of that finding is that, in females, it may be necessary to control not only for body size (height or BMI) as in men, but also for hormonal status in studies seeking associations with AGD measurements. In our study population, 74% (n = 29) of the young women taking hormonal contraception reported irregular menstrual cycles in the previous three months. However, no association was found between AGD measurements and irregular menstrual cycle. Therefore we could speculate that hormonal intake might be related to the AGD measurements. To our knowledge, this is the first time this association has been reported in humans.

Recently, Dusek and Bartos [31] examined the effect of the stage of the oestrous cycle on the AGD in female mice and showed that AGD varied during the oestrous cycle, and suggesting that female genital morphology systematically varied within the oestrous cycle. Unfortunately, all the AGD measures in our study were taken during the early follicular phase, so that we cannot assess that hypothesis. Nonetheless, it opens a door to the possibility that hormonal changes (including hormonal contraception) could result in small fluctuations in the morphology of the female external genitalia. Longitudinal study measuring AGD within women at multiple points in the cycles is needed to address this possibility.

Alternatively, it has been suggested that women using hormonal contraception are somewhat more fertile than women who do not [32]. In many mammalian species females with shorter AGD are more fertile [33,34]. Therefore, it is possible that the shorter AGD we observed in women using hormonal contraceptives reflects increased fertility, rather than a direct effect of hormone use on AGD.

As multiple animal studies have shown, the female reproductive tract is susceptible to virilization by exogenous androgen exposure prior to, as well as during, the in utero masculinization programming window (MPW) [5-7]. This prenatal hyperandrogenism results in enlarged cystic ovaries, anovulation or increases ovarian follicular recruitment in female offspring [3,4,7,35]. Excessive prenatal androgens (either endogenous or exogenous) can produce a polycystic ovary syndrome (PCOS)-like phenotype in animal models, furthermore [35,36]. As such, it has been widely hypothesized that the etiology of PCOS in humans may include excess prenatal androgen exposure [37]. Pregnant women with PCOS have elevated circulating concentrations of androgens at mid-gestation, which may increase fetal androgen exposure, and female offspring of PCOS mothers are at increased risk of altered ovarian development and function [38,39]. Long-term follow-up studies are required to determine whether this excessive exposure resulted in long-term changes in ovarian function and/or alterations in reproductive function. Here, we do not examine PCOS per se, however we do demonstrate that AGD, a purported biomarker of prenatal androgen exposure, is associated with increased ovarian follicle number.

With regard to reproducibility of follicle count estimated by transvaginal ultrasound, it has been shown that determination of follicle count by transvaginal ultrasound results in acceptable intra- and inter- observer variability [40]. While there is undoubtedly some inter-cycle variability of follicle count, it is considered to be of little clinical significance, for example, in predicting the response in in vitro fertilization [41]. Several articles have previously defined having ≥ 6 or > 12 ovarian follicles as multifollicular [28,29] or polycystic ovaries [30], respectively. We were unable to look at women with polycystic ovaries separately due to small numbers; only 10 women met this criterion. We chose instead to use the cutoff for multifollicular ovary (≥ 6), a cutoff which several studies suggest may have clinical relevance [28-30].

We found significant positive associations between both AGD_AF_ and AGD_AC_ and the presence of greater ovarian follicle number, which could indicate a common fetal origin between longer AGD and greater follicular recruitment. In fact, in animal models, an association between prenatal exposures to androgen excess and an increase in ovarian follicular recruitment [3,4,7] and longer AGD measurement [7] has been reported.

All previous published studies on human female infants have measured both AGD_AF_ and AGD_AC_[19,20,23,25-27]. Callegari et al. [23] also calculated the ratio (i.e.: AGD_AF_/AGD_AC_) both, in premature/full-term newborn infants and newborn infant girls with congenital adrenal hyperplasia, reporting a relatively higher ratio among subjects with that condition. This result supports the hypothesis that androgen exposure in utero may affect measures of the anogenital region in human females. Callegari et al. [23] also took both AGD measurements on a small number of pregnant women (n = 10), reporting the first such data, to the best of our knowledge. With all the possible limitations regarding study population and methodologies, our young women presented longer AGD_AF_ and shorter AGD_AC_ compared to those ten pregnant women.

We had only a few cases of clinically diagnosed PCOS in our study population; therefore we were unable to explore the relationship between that condition and the AGD measures. A larger and more diverse population or another type of study design (case–control) would add much more information on whether androgen action during early fetal life exerts a fundamental influence on the female reproductive tract in humans, as has been demonstrated in rodents.

Our population was small and limited in age and ethnicity, and thus cannot provide normative values for human female AGD measurements. AGD measures were well tolerated by all subjects, quick to perform, with acceptable intra- and inter-examiner reliability. We plan to assess reproductive hormones in a future publication. A finding of higher FSH and/or low inhibin-B, estradiol or free testosterone with shorter or longer AGD would lend support to the association between AGD and female reproductive system characteristics reported here.

In human males, shorter AGD in adulthood has been associated with poorer semen quality [21,22] and infertility [21] suggesting a common origin, including a disruption of testicular development in utero, as suggested by the testicular dysgenesis syndrome hypothesis [42,43]. As hypothesized, this syndrome, although potentially multifactorial, may be caused by exposure to EDC during the MPW [18].

Recently, Buck Louis et al. [44] suggested a similar paradigm for human females, the ovarian dysgenesis syndrome, which the authors define as alterations in ovarian structure or function that may manifest as fecundity impairments, gynecologic disorders, gravid diseases or later onset of adult diseases. Environmental exposures, particularly EDC, may be related to in utero origin of gynecologic outcomes, which in turn would be associated with later onset of adult diseases.

## Conclusions

Animal studies have shown that the female reproductive tract is susceptible to virilization by exogenous androgen exposure prior to, as well as during, the MPW that is determined by androgen action [6]. Our data demonstrate associations between the two measurements of AGD we examined and an increase in ovarian follicle number. Our results suggest, therefore, that the androgenic environment during early fetal life may exert a fundamental influence on both AGD and female reproductive system characteristics in humans.

If AGD (adjusted for body size) is determined prenatally in humans as in rodents, a longer female AGD in adulthood should reflect a longer AGD at birth, which in turn may reflect increased androgen exposure in utero. Therefore, both greater follicular recruitment and longer AGD in adulthood - assuming that AGD is a stable finding in humans throughout their lives - may reflect a common origin in utero, resulting in alterations of the developing female reproductive tract.

## Abbreviations

AGD: Anogenital distance; AGD_AC_: Anogenital Distance from the center of the anus to the anterior clitoral surface; AGD_AF_: Anogenital Distance from the center of the anus to the posterior fourchette; BMI: Body mass index; BPA: Bisphenol A; EDC: Endocrine-disrupting chemicals; MPW: Masculinization programming window; MYWS: Murcia Young Women’s Study; PCOS: Polycystic ovary syndrome; STD: Sexually transmitted diseases.

## Competing interests

The author(s) declared no conflicts of interest with respect to the authorship and/or publication of this article.

## Authors’ contributions

MR, JM, SHS and AMTC designed and initiated the current study. MR, LMA and JM were responsible for collecting the samples and the interview data. LMA and MR coordinated the current study. JJLE and MPME were responsible for statistical analysis. ESB, MPME, JM, MR, SHS and AMTC were responsible for writing the draft version of manuscript. All authors commented on and approved the final manuscript.
